# Sharing meals: promising nutritional interventions for primary health care including nursing students and elderly people

**DOI:** 10.1186/s40795-021-00412-8

**Published:** 2021-04-15

**Authors:** Ellen Kristine Frøyland Alne, Tove Øie, Malene Søiland, Kine Gjesdal

**Affiliations:** 1 Centre of Health Promotion, Stavanger, Norway; 2 Centre for Development of Institutional and Home Care Services, Stavanger, Rogaland Norway; 3 Faculty of Health Sciences, University of Stavanger, Stavanger, Norway

**Keywords:** Student nurse, Practical placement, Nutritional risk, Elderly, Community, Sharing a meal

## Abstract

**Background:**

The risk of malnutrition among elderly people is high and living alone increases the risk. As the number of older persons living alone is expected to increase due to the demographic development of an increasing older population, more knowledge about low-cost, sustainable nutritional interventions is needed. The purpose of this study was to investigate how nursing students can be a resource in the nutritional care of older persons living alone by sharing weekly meals.

**Methods:**

Twenty-three nursing students and 23 elderly people who lived alone and received home nursing care participated in the project period of 9 weeks and shared 1–2 weekly meals. Shortly after the study period, 13 students and 4 elderly persons were interviewed in individual, face-to-face, semi-structured interviews. The questions included their experiences, the perceived impact of sharing meals, and facilitators and barriers of such meal interventions. The interview material was transcribed and analyzed using qualitative content analysis as described by Graneheim and Lundman.

**Results:**

Our study found that both nursing students and older persons expressed positive experiences from sharing meals. Nursing students with some nutritional knowledge can provide a useful, sustainable supplement to the home-care nursing staff’s limited resources and time. Improvements were found, including preparation of ready meals and the meal environment, different facilitators and barriers of the meal experience, and the possible positive impact on the elderly persons’ nutritional status, which affects meal enjoyment, appetite, food intake and weight.

**Conclusion:**

During their practical placement in the community, nursing students can provide a useful contribution to the nutritional care of elderly persons who are at nutritional risk living alone at home by the intervention of sharing a meal together. This is a low-cost supplement to other primary health-care measures that can affect both nutritional status and adjust the appropriate care for patients. This study demonstrates a small contribution to the complex nutritional care literature based on the growing elderly population in home care and the nursing student as a valuable resource for the multidisciplinary team approach necessary to meet this challenge.

**Supplementary Information:**

The online version contains supplementary material available at 10.1186/s40795-021-00412-8.

## Background

Nutrition is an essential modulator of health and well-being in older persons [1, 2]. Nutritional intake is often compromised in older persons and many factors increase the risk of malnutrition [1, 2]. Malnutrition affects physical and psychological health and functioning, including delayed wound healing, impaired immune system, increased risk of pressure ulcers, muscle wasting and weakness, altered gastrointestinal structure and function, apathy and depression, a general sense of weakness and illness, and overall increased susceptibility to disease, impaired clinical outcomes, and increased health-care use and costs [3, 4].

The risk of malnutrition is highly prevalent among older people in the community. It is estimated to affect around 14.6–56% of older people receiving home care [5–7]. The frequency of malnutrition varies depending on the criteria used to define it: i.e. age, associated clinical conditions and treatment type [4]. The prevalence of malnutrition generally increases with deteriorating functional and health status [1, 2].

Studies have shown that undernourished older people were found to be twice as likely to experience poor quality of life than their normally nourished counterparts [8, 9]. Other studies have found that older people who report weight loss have a higher risk of developing limitations in activities of daily living (ADL), such as dressing, bathing and eating [10, 11] and that low body mass index (BMI) was a moderate risk factor for the onset of these limitations [9].

The loss of taste and smell is often associated with a decrease in food intake and is termed ‘anorexia of aging’ [1]. However, it may also be caused by poor oral health and difficulties chewing and swallowing. Other factors involved may be cognitive limitations, social isolation, depression as well as side effects of pharmacological treatment [1, 4].

Eating alone is independently associated with geriatric depressive symptoms [12]. Various acute and chronic medical conditions also increase energy needs and increase the risk of developing malnutrition in older people [1]. There may be other factors involved and the development of malnutrition is not fully understood [1].

The main aim of geriatric medicine is to optimize functional status and ensure the greatest possible autonomy and best possible quality of life for older persons [1, 2].

Oral nutrition is fundamental for pleasure and quality of life, not only by the provision of nutrients and enabling the sensations of taste and flavour, but also has significant psychological and social functions. It is of high priority to respect the patient’s wishes and preferences [1, 2].

Different methods can be used in attempts to improve older persons’ appetite. Improving the environment in which food is served could influence appetite. A systematic review of mealtime interventions in care homes found that improving the dining environment improved the residents’ weight status and food intake. These improvements included using nice crockery, tablecloths at mealtimes, improved choice of and access to food and mealtime assistance [13, 14].

Appetite is influenced by environment and mood. Depression is known to impair appetite and living and eating alone can cause reduced appetite. This may be caused by difficulties shopping and cooking, as well as fewer social cues and impaired motivation to eat [15]. It is encouraged to promote a social environment for older people with or at risk of malnutrition in order to increase food intake [16].

The current knowledge about the effectiveness of nutritional interventions is summarized in the European Society for Clinical Nutrition and Metabolism (ESPEN) guideline on clinical nutrition and hydration in geriatrics [2]. Our project contributes some key factors for suggested intervention strategies in the management of malnutrition, both as a nutritional intervention as part of a multimodal and multidisciplinary team intervention, sharing mealtimes with others, nutritional information and education, food fortification, increasing variety of food and considering individual preferences.

Eating with others is associated with subjective health, an increase in food intake and food enjoyment [12, 17, 18]. Individuals will probably also eat larger amounts if eating in a group rather than eating alone. The social impact of eating has been well documented with evidence from food diaries, and observational and experimental studies [18]. Therefore, this study aims to explore the following: What experiences do elderly people who live alone have by eating together with a nursing student with regards to their appetite, well-being, and meal enjoyment? What nutritional assessments and adjustments did the nursing students perform during this project?

## Methods

### Study design

This exploratory and descriptive study aimed to investigate experiences with a nutritional intervention in elderly people living alone who receive home nursing care. An exploratory design is used when little is known about the phenomenon to provide in-depth knowledge and a more nuanced understanding. This descriptive section attempts to present the issues precisely.

### The intervention

The sharing meals intervention was a collaborative project between a Centre for development of Institutional and Home Care Services, the Nursing Education Department at the University of Stavanger, a Centre of Health Promotion, and a municipal health service in Rogaland, a Norwegian region. The intervention corresponded with a regional and national initiative, called “A full life – all your life”, which is an initiative for older persons to reform their quality of life [19]. The goal of the initiative is for municipality residents to receive services that support them in being able to live a full life all their lives. This means that they will receive health-care services in a way that supports the residents to live an active life based on their resources and opportunities.

In the sharing meals intervention, a nursing student were paired with an elderly person who lived alone and was at risk for malnutrition. The food was ordered using a local home-delivery meal service for elderly people and required heating. The food was mainly traditional Norwegian food on a rotational menu that changed every 5 weeks. The municipality covered the expenses for the food in this project for both the nursing students and the elderly persons. The student was at the elderly person’s home to cook and eat together 1–2 times per week during the nursing student’s clinical placement of 9 weeks.

Thus, the sharing meals interventions began in February and August 2019 because these months were when nursing students began their practical placements in home care. The nursing students were all in their fourth or fifth semester of a Bachelor of Science degree in nursing and in one of their six periods of 9-week clinical placements. Norwegian nursing education covers basic nutritional competence. In preparation for and during their participation in the intervention, however, all students underwent additional education with different specialists in geriatric nutrition arranged by the municipality and the university. This was done to ensure their competence regarding nutritional risk in older persons and thereby increase the quality of the sharing meals intervention by adjustments of the meals and food fortification (Table 1).

**Table 1:** Students’ additional geriatric nutrition education

Examples of student learning strategies during practice	Academic contents
Information and education (2 days)	Malnutrition: prevalence, risk and management, communication skills, oral health and meal environment.
Online course (2 h)	Basic nutrition digital program for health personnel focusing on malnutrition.
Observation and assessment document	Enhance older persons’ self-determination and investigate what they consider important. Investigate measured weight, height, BMI, weight history, appetite, food intake, Mini Universal Screening Tool (for nutritional risk)
Self-reporting nutritional knowledge	Assessment of students’ knowledge about nutrition, malnutrition and older persons.
Reflection paper on nutrition	Reflection of students’ own experiences in sharing a meal.

The elderly participants fulfilled the following criteria: they were living alone and had different measures related to being at risk of malnutrition, e.g. help preparing food and regular weight and food intake monitoring. The elderly female and male participants were all aged between 60 and 93 years old with a BMI from 17 to 28 kg/m^2^.

### Data collection

**Fig. 1:**
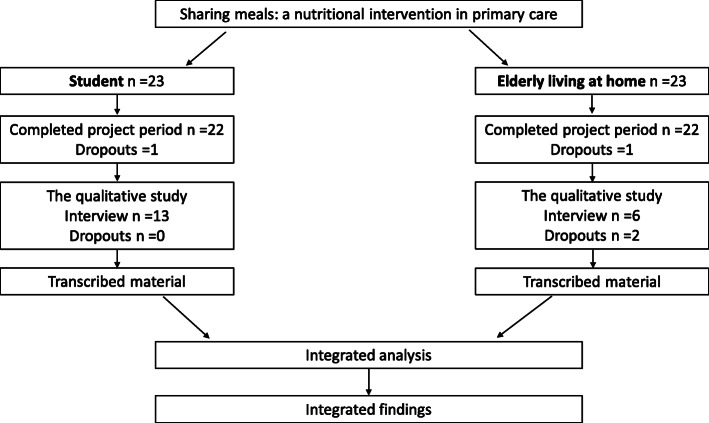
Illustration of the nutritional intervention and following qualitative study

Twenty-three nursing students and 23 elderly people in need of nutritional care participated in the sharing meals intervention. We invited all students and elderly participants in the intervention to participate in the study to share their experiences. Of these, 13 students and six elderly persons accepted this invitation. However, two elderly persons were excluded from the study: one due to recently discovered cognitive impairment and one due to worsened medical condition (Fig. 1). The interviews were conducted individually to avoid conditional responses.

Thus, 13 students (12 females, 1 male) and 4 elderly participants (all female) were interviewed individually. We interviewed the elderly at home and the students in a suitable and quiet room at the university. This was done shortly following the placement at the end of the intervention. The semi-structured interviews consisted of questions about the practical feasibility of the project, the experience of eating together and the possible effect on quality of life, food intake and nutritional status (Table 2 and supplementary file). The interviews lasted 45–90 min, depending on how much they wanted to elaborate around the different themes.

**Table 2:** Overview indicating the interview guide for the nursing students and elderly participants

Example of questions for the student	Examples of questions for the elderly participants
*What are your experiences with this project of sharing a meal?*	*What are your experiences being a host?*
*In what ways did you learn about nutrition through the project?*	*How did you contribute to the choice and preparation of the food?*
*Can you tell us about how you planned and organized the meal?*	*In what ways did the student assist and inform you?*

### Data analysis

The transcribed material was analyzed using qualitative content analysis as described by Graneheim and Lundman [20]. The stages of the analytical process were as follows: open reading, where all co-writers read each script several times to get an idea of what was said. Then, the first author identified patterns of the data by dividing the text into meaning units and condensing them into a more formal and written style. Next, the first author created codes for labelling. Comparing the codes based on differences and similarities, as well as sorting them into categories, was performed in collaboration with all co-authors. Tentative categories and themes were discussed and formulated with a manifest content (Table 3). We created joint categories to find similarities from each question set around the different themes to get both students’ and the elderly persons’ view across.

**Table 3:** Example of the abstraction process from meaning units to themes

Meaning unit	Category	Theme
*We put the food on plates. It looked more beautiful.*	Mealtime environment	Improved meal preparation
*We had two portions of the same meal and put the food on serving plates. Then we could help ourselves at the table and the portion would be smaller when appetite is poor.*	Food and nutritional tailoring	
*I was very lucky; we had good chemistry and it was all very nice. I looked forward to it and thought this is going to be nice. I tried to think: What can be good for this patient?*	Surroundings affecting the meal	
*I thought about fortification and energy-dense food, and I talked to the dependents about it... and it helped because her weight increased.*	Nutrition-related factors	Mixed meal experiences
*My host always talked about how nice it is to eat together. We experienced that it was very useful for her and a good thing.*	Facilitators for a good meal	
*The host liked to have a visit, but it was difficult that the visit was about food. She thought the visit was nice, and she is very social, but she didn’t manage to eat together with me.*	Barriers for a good meal	
*She has eaten very little because she has been lonely. Just eating with someone helped her a lot because she ate the whole portion when I was there.*	Appetite and enjoyment of the meal	Significant experiences contributing to improved nutritional status
*I noticed when we talked, he didn’t notice that he eats and then he eats more.*	Food intake	
*I worked together with the patient’s nurse and documented the different measures.*	Multidisciplinary nutritional care	

## Results

This study explored the experiences from a shared-meal intervention including nursing students and elderly people living at home receiving home care. Our findings are presented according to the following themes: “improved meal preparation”, “mixed meal experiences” and “significant experiences contributing to improved nutritional status”. The quotes are numbered and marked with an S (student) or an E (elderly) to clarify the sender.

### Improved meal preparation

Improved meal preparation consists of important factors involving the meal surroundings and the facilitation and tailoring of the meal and environment. Both students and elderly participants were involved.

### Mealtime environment

There were many examples of students and elderly participants making small adjustments to set a nice table and present the food in a beautiful way. Each of the participant pairs solved this differently. Some students performed these activities on their own, while some elderly persons prepared their home environment alone prior to the shared meal, and in some situations, the students and elderly participants did this together. The meals used in this project were ready-made meals that required heating:

*We eat a meal and facilitate … and make sure her sitting position is good. We don’t just say “hi, let’s eat”, but we think about the meal environment as well. S#8*

We found that the students and elderly participants improved the appearance of their meals by putting the food on nice plates and in bowls. They thought this was more beautiful:

*The first time we put the food directly on the plate, but then we experienced that it was better to put the food in trays and serve ourselves. The first time we did it, she only had two meatballs and a spoonful of mashed potatoes. I thought: “Is this all she is having?” (laughter) But then she helped herself three times because she liked it so much, which was very nice. It showed that she wanted more food when we sat and talked, and she could help herself.* S#9

Many elderly participants also used tablecloths, napkins, lit candles and decorated the table with flowers to make the food and meal situation more appetizing:

*We turned off the radio when we ate. And she had already put out some nice napkins and a tablecloth and some flowers. The meals were very pleasant.* S#4

*We did it together. I think she experienced coping and a feeling of having a guest in the house by setting the table.* S#9

### Food and nutrition tailoring

Food and nutritional tailoring are important to adjust the meals and food to the various individuals’ needs. Although nutritious and tasteful, the ready-made meals were not fortified or energy dense. Ready-made meals have limited possibilities for individualization, but many students nevertheless managed to adjust the meals according to the needs of the elderly persons in various ways, e.g. by improving the energy and protein content and adjusting the portion size. The nursing students ordered the same meal as the elderly participants they were paired with to be able to mutually share the taste experience, and the students chose food that they thought the elderly person would be familiar with when they experienced difficulties choosing a meal from the menu:

*I ordered the same food for her and me for us to have something in common. It is nice to talk about the food when we eat the same meal.* S#10

*I think it is useful for her, me being there eating with her. Because then we do something together.* S#3

Many nursing students helped the elderly persons with smaller portions of food when their appetite was poor. Smaller portions can be more appetizing for elderly persons with a decreased appetite:

*Some days her appetite was poor. I said: “we can share a portion, you and I?” Then she ate a small portion. It was not ideal, but better than nothing.* S#3

*We had two portions of the same meal and put the food on serving plates. Then we could help ourselves at the table and the portion would be smaller when appetite is poor.* S#1

The students also managed to fortify the meals using extra butter, oils, cheese and cream where appropriate. To enhance the elderly persons’ nutritional intake, the students also provided an additional dessert. When the elderly persons had decreased taste sensation, the student used additional herbs and spices with the meal to improve the elderly persons’ taste experience:

*But of course, we put extra butter in the mashed potatoes, little things that fortify the food.* S#9

Many of the elderly participants were used to eat ready meals and were involved in the fortification of the food. The dessert was a positive contribution and improved the nutritional and energy content of the meal:

*The elderly woman liked something sweet following the meal. The last day, I brought donuts that we ate. She liked that a lot.* S#3

*She really appreciated the dessert. She did not always finish the main meal. She put it aside and saved it for the evening, but she always managed to finish the dessert.* S#10

### Environment affecting the meal

We found that the students tried to create a good atmosphere for sharing a nice meal together with their elderly partner. Many students were concerned about empowering the elderly participants and tried to explore what mattered to them:

*I was very lucky. We had a good chemistry and it was all very nice. I looked forward to the meal and thought this is going to be nice. I tried to think: “What can be good for this patient?”* S#3

The students reported that the elderly participants expressed increased coping by preparing a meal together. However, many students experienced that the elderly participants were content when the students took care of all the practical needs around the meal:

*To her, it was important to set the table nicely. I was a guest in her house, and she wanted everything to be nice and well presented.* S#9

*I can’t manage a lot, but I think I could have done more than I did, but he took care of everything.* E#3

### Mixed meal experiences

This theme contains experiences relating to mixed meal outcomes and the different influences of meals such as nutrition-related factors and the facilitators and barriers to a good meal.

### Nutrition-related factors

Some students reported the meal as an opportunity to talk to the elderly participant about nutrition while others reported the opposite, i.e. the meal was primarily a good arena for social interaction and to a lesser extent giving direct nutritional advice. Some elderly participants agreed that combining eating and nutritional advice would be challenging:

*I thought about fortification and energy-dense food, and I talked to the dependents about it.... and it helped because her weight increased.* S#3

*We talked about what food she liked, and what food she was used to eat. And we talked about her difficulties eating and her lack of appetite. But it didn’t feel natural for me to tell her: “you must eat this and this is important.” This pressure would destroy the nice meal situation that I tried to create.* S#1

*The conversation flowed easily. It was not a problem. We talked a lot about social things and talked a lot less about nutrition.* S#4

*We talked about everything. It felt like we’d known each other our whole life.* E#3

*If a person came to me, to eat with me and talk about nutrition, I wouldn’t like that.* E#1

Nevertheless, the students demonstrated other indirect ways in which they used their obtained nutritional knowledge through food fortification, considerations related to existing medical conditions and catching up on inappropriate situations and equipment used by the elderly persons during the meals:

*And he has regular plates, but I noticed that when he eats, he pushes the food to the edges of the plate. And then he has problems getting the food on to the fork. So now I made them (home-care nursing staff) order special plates to make this easier. … I do not understand why no one has thought about it already.* S#11

Eating and preparing a meal together gave the students increased valuable insight into the situation of the elderly participants compared with the possibilities for the home-care nursing staff with their limited time and resources.

### Facilitators and barriers to a good meal

Eating together can be an intimate, personal experience. The nursing students and elderly participants had many perspectives on the facilitators of a good meal. They all had enough time during each visit, which was important for getting to know each other and creating a nice atmosphere. Most students and elderly participants appreciated this moment together. They talked a lot about many different subjects, and the students said they felt the elderly patients enjoyed the conversations as well as the food:

*It was a nice way to take care of more than just their physical needs because we had one hour where we could talk together, and in that way, we could take care of both social and psychological needs as well.* S#1

*My patient always talked about how nice it is to eat together. We experienced that it was very useful for her, and a good thing.* S#2

*I thought it was very interesting, I got varied meals, and I got to meet a nice student. We hit it off immediately, and I looked forward to every time he was coming.* E#3

*I said to her: “Visit me when you come back, if you are not too busy with your boyfriend” (laughter). Nice girl, I enjoyed it a lot when she came.* E#4

The students mentioned some important barriers. One student said they had to end the project because the elderly person had a complicated relationship with food and did not look forward to eating with someone:

*The elderly person liked to be visited but found it difficult that the visit was about food. She thought the visit was nice, and she is very social, but she could not manage to eat together with me.* S#1

The students mentioned different barriers to a good meal. Some homes were challenging for creating a good appetite and meal due to hygienic factors, and some students felt that it was a difficult experience to enter a private home and eat together with someone they did not know. Others recognized the elderly participants as being lonely but felt that they needed more activities and to get out of the house instead of sharing a meal. Some participants described the intervention period as being too short to be able to create a good relationship:

*I worried about what we were going to talk about. Did she have clean plates? What is the cutlery like? I worry about all these things.* S#1

*I think they (home-care nursing staff) were a bit sceptical about the project. I heard that. But I understand. It is not always easy to let a stranger into the house and share a meal.* S#13

### Significant experiences contributing to improved nutritional status

Several factors may contribute to improved nutritional status. We divided our findings into appetite and meal enjoyment, food intake and multidisciplinary nutritional care.

### Appetite and meal enjoyment

Mealtimes are when many elderly persons felt most lonely and many had difficulties eating alone. The nursing students reported that their visits and the shared meals affected the elderly persons’ moods positively and was something they looked forward to. Students said the home-care nursing staff also noticed that the meal and visit evidently affected the elderly persons’ mood both before and after each meal. This was reported by both students and elderly:

*She has eaten very little because she has been lonely. Just eating with someone helped her a lot because she ate the whole portion when I was there.* S#4

*I want to get across that this is a project I hope can last, really. I have a great impression of it and when I see how happy the patient is when I stay there, for as little as an hour maybe.* S#2

*I think it is very positive to have someone to share a meal with. I have problems eating alone, to me it is very good to eat together because then I manage to eat.* E#1

The students reported various other possible factors influencing appetite and food intake. The food smelled good and many elderly participants clearly enjoyed the prepared food:

*It smelled good both in the kitchen and in the living room. That might have stimulated the senses.* S#4

*It was easier to eat when I did not use all my strength in preparing the meal.* E#1

### Food intake

Sharing meals may have affected food intake in many of the elderly participants because many of them mentioned it as a factor that affected their food intake. Many students reported that their elderly patients ate the whole portion of food and some dessert despite having difficulties eating:

*I noticed when we talked, he didn’t notice that he eats and then he eats more.* S#6

*She enjoyed the meal and ate all the food. Even though the portions were very big, she ate everything, which was surprising.* S#5

In addition to improved appetite and enhanced food intake in the elderly, many students reported that their elderly patients gained weight. This was probably due to a combination of different measures in nutritional care:

*She had lost some weight previously and then her weight increased by 1.5 kg.* S#4

*I think it was a combination of sharing a meal and other measures that increased her weight.* S#2

### Multidisciplinary nutritional care

Older patients’ nutritional care consists of many different factors and is most successful with a multidisciplinary team approach. The contribution of the nursing students was valuable. The students interacted with the patients and informed the home-care nursing staff of their observations. This resulted in the continuously improving and better tailored care of the patients:

*I found it difficult to change anything because everything is controlled by the measures of the home-care nursing staff and the time allocated. But I had many good conversations with the other nurses around the nutritional situation of the patient.* S#1

*There was no weight documented, even though there was a danger of malnutrition.* S#9

The increased care around the meal situation was also appreciated by many of the elderly participants:

*It is nice that someone came to eat together with me instead of home-care nursing staff who just make sure I get enough food.* E#1

The elderly participants had experiences of the home-care nursing staff’s time constraints making co-eating not feasible.

## Discussion

We found that both the nursing students and elderly participants enjoyed their meals together, but they needed time to get to know each other. Eating together creates an important social arena and can influence well-being as well as appetite and food intake [17, 18]. However, food intake, eating and the elderly person’s relationship with food and eating can be complex [21, 22]. There were many examples in our findings of the shared enjoyment of the meals together; however, some elderly participants felt that the experience was invasive and wanted to withdraw from the project. Respecting both elderly persons and students in the complexity of sharing meals as well as combining elderly persons and students that get along is important. Perhaps different pairings based on personal criteria could be adopted for future projects.

Many of the elderly participants were feeling alone and this contributed to a self-reported loss of appetite in our study. Eating together is an important way to improve appetite and food intake, and many elderly participants experienced improved food intake, appetite, and mood. This finding is in accordance with research exploring the importance of eating together [17, 18, 22]. Being social and interacting with both family and friends is of great importance, both for healthy eating habits and maintaining good psychological health [23].

We also found that students were concerned about adjusting the experience of sharing a meal in accordance with what was important to the elderly participants. This type of person-centred approach can help establish a relationship and is also helpful to understand the person in the context of his/her own life and the things that are most important to them [24]. We know that due to current population demographics, more older people are living longer at home and the disease burden has increased and will continue to increase [16, 19, 25]. To give the best quality of care and provide the correct amount of assistance and resources, we found that sharing meals was valuable due to the setting and the time that the student and the patient spent together. However, a close liaison with the home-care nursing staff is necessary to obtain this quality of care.

The nursing students participating in the sharing meals intervention provided a useful contribution to individualizing ready-made meals with their suggestions of ways to improve the appearance and the nutritional content in collaboration with their elderly patients to enhance their food intake. The nutritional content of the meals was standardized and nutritious, but not specifically energy- and protein-rich. This could be improved by specifically tailoring the food ordering and delivery service to the nutritional needs of the elderly patients [26, 27]. However, this type of food service was not available during our project. To adjust the food according to the needs of the elderly patients, the student’s nutritional knowledge and their collaboration with the home-care nursing staff was crucial.

Studies have shown that appropriate nutrition intervention for home-delivered meal (HDM) participants can improve their health condition and delay chronic diseases. HDM participants must be a primary focus in more effective nutrition education and counselling [28]. However, we found conflicting evidence as to whether sharing meals was a good opportunity for the students to talk to their patients about nutrition, provide basic nutritional information to elderly persons and make any nutritional assessment. Both students and elderly participants experienced the meals primarily as a social interaction and to a lesser extent an opportunity to assess, give and receive nutritional information. There might be several reasons for these findings related to both the setting, the elderly participants’ needs, and the qualifications of the students compared with fully qualified nurses and other health-care personnel. Dietitians are qualified and regulated health-care professionals who can assess, diagnose, and treat nutritional problems. They translate nutrition science into practical guidance to enable people to make appropriate lifestyle and food choices [29]. There is a need for the increased availability of clinical nutrition expertise in Norway, especially in municipalities [30]. Nevertheless, this study indicates the importance of student nutritional knowledge in this intervention to make important adjustments, pick up on nutrition-related factors and collaborate closely with the home-care staff to improve overall patient care.

There is considerable evidence to recommend routinely screening for malnutrition in elderly patients in the health-care services, using a validated screening tool [31]. Screening for malnutrition was also recommended in this study, but we found that this was only done in exceptional cases. The lack of focus on nutrition and documentation was also shown in several other studies [32, 33]. There might be various reasons for this: e.g. sometimes there is no opportunity to ask about or observe food intake, to measure patient weights or screen for nutritional status in the time allocated [34, 35]. The lack of nutritional screening is in accordance with the findings of the state’s health supervision in Norway where 80% of Norwegian municipalities did not perform systematic screening for malnutrition [32]. More knowledge and systematic work with nutritional care in home-care services in Norway is recommended.

This study makes a small contribution to the complex nutritional care literature focusing on the growing elderly population in home care and the student as a valuable resource in the multidisciplinary team approach necessary to meet this challenge. Involving nursing students or other student health-care personnel will make a sustainable contribution to the complex management of malnutrition, which is expected to increase in future with the increasing number of frail elderly persons living in the community [16]. The nursing students obtain valuable nutritional knowledge that they can benefit from in their professional life and the elderly obtain an important social arena that possibly positively affects their nutritional risk.

### Strengths and limitations

The strengths of this study are the achievable and inexpensive supplements to nutritional care for elderly patients by sharing meals with nursing students during their clinical placement. Thus, the intervention can be a low-threshold nutritional opportunity for elderly patients at nutritional risk. Simultaneously, it can be a valuable learning opportunity for the nursing students. In addition, the intervention is easily to adjust and easily replicable for other municipalities that want to implement the sharing meals intervention. However, this is a small study where not all participants in the intervention wanted, or were able to, be interviewed and share their experiences. The sharing meal project was one (of several) important measures the municipality offered the elderly at nutritional risk, and for ethical and nutritional reasons we could not exclude them from the project even if they would not be interviewed afterwards. The reason why only a few of the elderly agreed to be interviewed is uncertain, as they did not have to state the reason.

Most nursing students at this course were female and because of this, most participants were female. The elderly were all female. This might have affected the result in the sense of the female perspective. However, we have no reason to believe that this is a project mostly suited for a specific gender.

There is a lack of quantitative data on the effect of the intervention. More research is needed on this type of intervention. An important point is that the study includes the important voice of vulnerable elderly people at nutritional risk, who are rarely involved in qualitative research studies. To ensure the sustainability of the sharing meals intervention, future projects may consider the use of volunteers to maintain these important social meals over an extended period.

## Conclusions

The study findings support the importance of continuous attention to the risk of malnutrition in primary health care. Our study demonstrated positive experiences for both students and elderly participants by sharing meals together, when other meal interventions, such as meals at community senior centres, are not applicable. This study implicates a valuable, sustainable, low-cost and social contribution to the complex nutritional care of isolated elderly persons. It gives an important voice to elderly persons living at home to ensure high and correct quality of care.

Our findings show promising qualitative results, but more qualitative and quantitative research studies are needed to further investigate the effect of the sharing meals intervention on nutritional risk, nutritional status, appetite, and quality of life of elderly persons living at home.

## Supplementary Information

**Additional file 1.** Interview guide - Sharing meals.

## Data Availability

The datasets analysed during the current study are not available. According to the ethical approval, only the members of the research team are granted access to these data sets.

## Abbreviations

ADL: Activities of daily living
BMI: Body mass index
S#1: Student interview no. 1
E#2: Elderly interview no. 2
