# Supplementary material for: Sharing meals: promising nutritional interventions for primary health care including nursing students and elderly people
Source: BMC Nutr. 2021 Apr 15;7:8. doi: 10.1186/s40795-021-00412-8 (PMC8048158; doi:10.1186/s40795-021-00412-8)
Supplement: Supplementary file 1 — Additional file 1. Interview guide - Sharing meals. [file 40795_2021_412_MOESM1_ESM.docx]

**Interview guide - Sharing meals:**

***Student:***

How do you understand the concept, sharing a meal, in this project?

What are your experiences with sharing a meal with an older person as part of your practical placement?

How did you experience getting to know the older person?

How did you experience communicating with the older person?

How did you experience learning about nutrition and malnutrition by participating in the project?

What would you say influenced your nutritional knowledge and education in the project?

- In what ways did you benefit from the additional education in geriatric nutrition?
- What would say hampered your nutritional knowledge and education in the project?

How did you experience working with the older person (preparing the meal)?

-Who performed the various tasks during the preparation of the meal?

- Can you give examples of what the food looked like and what the meal environment was like?

- Is there anything we have not talked about that you want to express?

***Older person:***

Can you tell me about your experiences participating in this project?

If you had a choice, how many times a week would you share a meal like this?

Did you get to know the student?

How did you experience the communication with the student?

What are your experiences of working with the student? (preparing the meal)

In what ways could you influence the choice of food?

In what ways did you participate in the preparation of the food?

Can you tell us more about who did what?

What do think about the portions of food and the taste of the food?

In what way did you get help and guidance from the student?

What are your experiences with eating with a student**?**

Did you experience any effect on your appetite when eating with the student?

Was the time of the visit and the meal suitable for you?

- Is there anything we haven’t talked about that you wish to express?
